# Metastatic prostate adenocarcinoma presenting with pulmonary symptoms: a case report and review of the literature

**DOI:** 10.1186/1757-1626-1-316

**Published:** 2008-11-17

**Authors:** Moustapha Tohfe, Samah Abdel Baki, Wissam Saliba, Fatmeh Ghandour, Raja Ashou, Georges Ghazal, Joudy Bahous, Nabil Chamseddine

**Affiliations:** 1 Hematology-Oncology Division, Faculty of Medicine University of Balamand, Saint George university Hospital, Beirut, Lebanon; 2 Department of Pathology, Faculty of Medicine University of Balamand, Saint George university Hospital, Beirut, Lebanon; 3 Department of Radiology, Faculty of Medicine University of Balamand, Saint George university Hospital, Beirut, Lebanon; 4 Urology Division, Faculty of Medicine University of Balamand, Saint George university Hospital, Beirut, Lebanon; 5 Pulmonary Division, Faculty of Medicine University of Balamand, Saint George university Hospital, Beirut, Lebanon

## Abstract

**Introduction:**

Prostate cancer has a high tendency to spread to bone. Pulmonary metastasis and generalized lymphadenopathy commonly develop after pelvic and bone involvement have already occurred. Few patients with prostate cancer present initially with symptomatic metastatic lung lesions and lymphadenopathy without any other concomitant distant dissemination.

**Case presentation:**

We report a case of a 73-year-old white male who sought medical help for symptoms of cough, hemoptysis, and dyspnea. A chest X-ray was done revealing multiple "cannon ball" infiltrates involving all segments of the lung parenchyma. Fine-needle aspiration cytology under computed tomography guidance of a subpleural lesion revealed adenocarcinomatous cells. Despite the absence of any detectable osseous lesions and with the presence of multiple hilar, mediastinal, para-aortic, and pelvic lymphadenopathy, the patient had a complete work-up in search for the primary adenocarcinoma. His prostate specific antigen was 146 ng/ml and a prostatic biopsy done, revealing an acinar prostatic adenocarcinoma. A tru-cut biopsy of a lung lesion under computed tomography guidance showed a metastatic prostatic adenocarcinoma positive for prostate specific antigen stain.

**Conclusion:**

This case sheds light on an unusual metastatic pattern of prostatic adenocarcinoma. It also emphasizes the importance of including prostate cancer in the differential diagnosis of men with adenocarcinoma of unknown origin.

## Introduction

Few reports in the literature have described an unusual pattern of presentation of metastatic prostate cancer. Pulmonary metastases are not encountered commonly in patients with prostate cancer. We report a case of a patient with prostatic carcinoma, whose pulmonary manifestations were the only evidence of his disseminated disease [1-3].

The patient had pulmonary metastatic lesions, rather than the conventional pattern of osseous metastases with subsequent pulmonary involvement commonly seen in patients with prostate adenocarcinoma. His pulmonary symptoms were not associated with other symptoms pertinent to a primary prostate neoplasia including lower urinary tract symptoms. Androgen deprivation remains an effective treatment and, among hormone-naïve patients, objective response is common [2].

## Case presentation

A 73-year-old white man with no relevant past medical or surgical histories, presented with symptoms of cough, progressive dyspnea, and two episodes of hemoptysis, not associated with any fever, weight loss, or night sweats. The patient did not report any symptom suggestive of urinary obstruction such as hesitancy, intermittency, weak stream, dribbling, or incomplete void. He was referred by his family physician to a pulmonologist, who in turn asked for a complete work-up including a chest X-ray, complete blood count, and a serum biochemical evaluation. The patient's laboratory evaluation was within normal ranges with a creatinine of 1 mg/dl (N<1.3 mg/dl), alkaline phosphatase of 75 U/L (N<104 U/L), and no evidence of anemia, leukocytosis, hypoalbuminemia, electrolyte imbalance or disturbed liver function tests. His X-ray film showed "extensive cannon-ball type lesions" involving both lungs.

**Figure 1:**
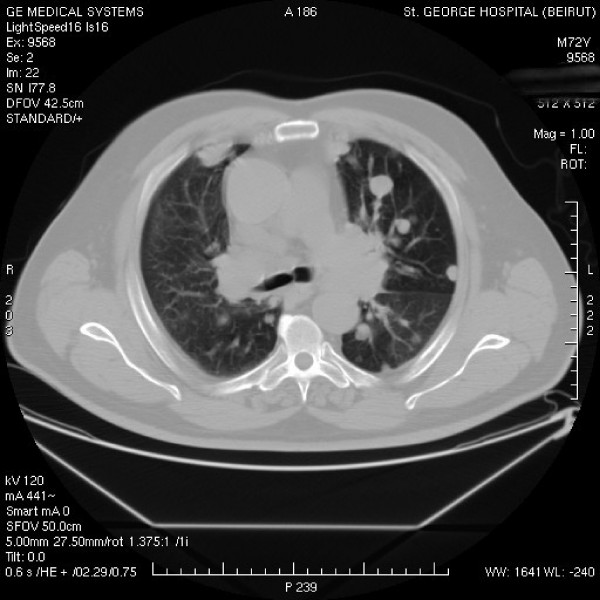
**CT-scan taken at the level of the carina**. Multiple metastatic nodules evolving all the lung segments with bilateral enlarged hilar lymph nodes.

The patient underwent an enhanced computed tomography (CT)-scan of the chest, abdomen, and pelvis. The CT-scan of chest showed a diffuse nodular type of metastatic disease involving all segments, with multiple pleural based lesions ranging in size from few millimeters to 2 cm (Figure 1). There was also evidence of multiple mediastinal enlarged lymph nodes, the largest measuring 5.3 × 3.2 cm at the level of the left upper lobe bronchus. Few lymph nodes were also noted in both axillae. No gross bony lesions were observed. CT scan of the abdomen and pelvis revealed multiple retrocrural, para-aortic, and pelvic lymph nodes with no lesions seen in the liver, spleen, pancreas, and adrenals. The patient had no ascites but had an enlarged prostate with a volume equivalent to about 77 g.

The presence of the above described lesions of the lung and enlarged lymph nodes raised the possibility of either a primary lung cancer with diffuse metastases or a lymphoma.

Fine needle aspiration cytology of a subpleural lesion under CT-guidance was suggestive of a non-small cell carcinoma that could probably be arising from an adenocarcinoma. The patient was asked to perform a series of tests including an assessment of his prostate specific antigen (PSA) which in turn was 146 ng/ml (N<4 ng/ml). His high PSA was associated with the presence of a nodular left prostatic lobe on digital rectal examination. A prostatic biopsy revealed a moderately differentiated acinar prostatic adenocarcinoma with a Gleason's score of 9 (4+5)/10. A whole-body bone scan revealed no evidence of bone metastases.

**Figure 2:**
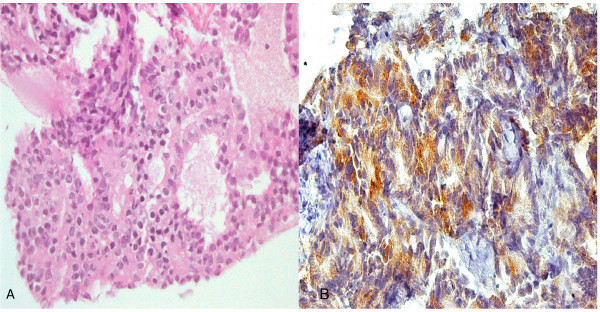
**A- Lung mass biopsy showing moderately differentiated adenocarcinoma, medium power.** B- Inset: PSA immunohistochemical stain of tumor cells are strongly positive.

A tru-cut biopsy of the lung under CT-guidance showed a metastatic prostatic adenocarcinoma, identical to the prostatic biopsy and positive on immunohistochemistry for PSA and negative for cytokeratin 7 (Figure 2).

**Figure 3:**
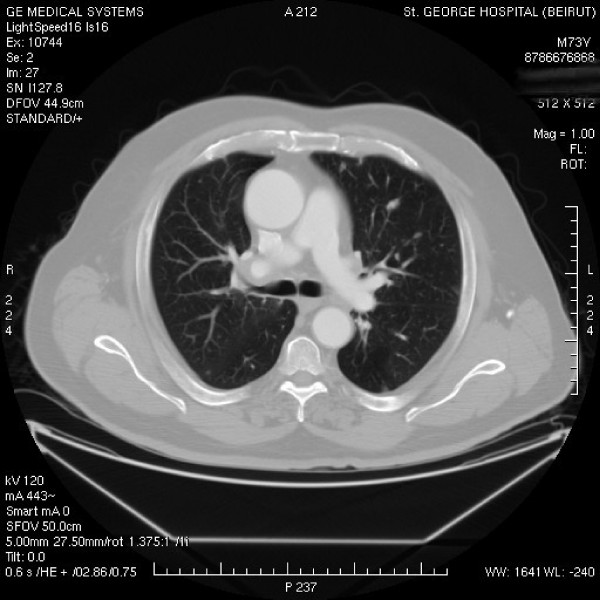
**CT-scan taken at the level of the carina**. Remarkable decrease in size and number of the nodular metastases as well as of the hilar lymph nodes.

The patient was commenced on total androgen blockade consisting of lutenizing hormone releasing hormone analogue and nilutamide (anti-androgen). Three months after therapy, PSA dropped to 0.19 ng/ml (N<4 ng/ml) and a restaging chest CT-scan was done revealing a remarkable decrease in the diffuse metastatic nodular lung disease as well as in the mediastinal lymph nodes (Figure 3).

## Discussion

Prostate cancer accounts for about 25% of new cancers in men [1]. The lungs are second or third only to bone and/or lymph nodes as metastatic sites from prostate cancer [4,5]. The reported incidence of clinically apparent pulmonary metastases at initial diagnosis is 5–27% [2], whereas autopsy rates show an incidence of between 23 and 74% [4,5]. Pulmonary lesions were part of the initial pattern of metastases in 2% of the population and developed subsequent to other metastatic sites in 1.6% of the population [2].

In this report, we document a case of prostate cancer where pulmonary metastases and lymphadenopathy occurred at presentation without any concomitant osseous or multiorgan involvement [3]. Radiological profiles of pulmonary metastases in advanced prostate cancer are commonly either lymphangitic or nodular, with the former being more frequent since it is apparently a result of direct invasion of lung lymphatics, while the nodular pattern being the consequence of hematogenous spread [2,4]. This patient had a nodular pattern on CT which is usually associated with other forms of hematogenous spread including bone involvement which was not evident in our case [6,7]. As a result, our patient seems to have developed metastatic nodular disease because of hematogenous spread, without developing bone metastases. This pattern of spread opens the door for more research venues about the pathophysiology of the mechanisms underlying distant metastases in prostate adenocarcinoma.

Classically, two lymph node metastatic patterns were suggested in prostate cancer: Pattern 1 includes metastases to pelvic and para-aortic lymph nodes, while pattern 2 includes metastases to para-aortic nodes only. In Type 1 cases the lymph node metastases appeared to be continuously invasive and significantly associated with less frequent metastases to the lungs. The Type 2 lymph node metastases may be a "skip-type metastases" or lymph node metastases associated with hematogenous metastases [5]. Our patient has most probably developed a hematogenous spread of his prostatic cancer cells to both lymph nodes and lung parenchyma [5]. Another major feature which makes this patient unique in presentation and difficult to diagnose is the presence of distant metastases and lymphadenopathy in the absence of bone involvement. The above mentioned clinical findings are relatively uncommon upon initial diagnosis [8] and the absence of urinary symptoms might mask the suspicion of prostate cancer as the primary adenocarcinoma that mimics a metastatic lung cancer or a lymphoma [9]. The rapid and dramatic regression of the lung lesions and of the lymph nodes confirms their metastatic nature and shows androgen deprivation to be an effective mode of palliation even for metastatic prostate cancer without bone involvement.

## Conclusion

This report emphasizes the importance of suspecting prostate cancer in men presenting with secondary lesions in the lung even in the absence of bone metastases. More clinical and basic research is needed to understand the molecular profile, epidemiology, and treatment options and responses of patients with prostate adenocarcinoma that present with lung metastases in the absence of bone involvement. In this patient, hormonal therapy for prostate cancer was also shown to be effective as first line treatment [2,5,8].

## Abbreviations

CT: computed tomography; PSA: prostate specific antigen.

## Consent

Written informed consent was obtained from the patient for publication of this case report and accompanying images. A copy of the written consent is available for review by the Editor-in-Chief of this journal.

## Competing interests

The authors declare that they have no competing interests.

## Authors' contributions

MT and SAB participated in the literature review and wrote the manuscript. FG was the pathologist who examined the biopsies. RA was the radiologist who read the CT and performed the biopsy. MT, WS, GG, JB and NC participated in the management of the patient. All authors read and approved the manuscript.
